# Prevalence of Obstructive Sleep Apnea in Head and Neck Squamous Cell Carcinoma Patients before and after Treatment

**DOI:** 10.3390/medicina60020310

**Published:** 2024-02-11

**Authors:** Olaf Gil, Benjamin Fenske, Thomas Bremert, Marcus Vollmer, Christian Scharf, Chia-Jung Busch, Markus Blaurock

**Affiliations:** 1 Department of Otorhinolaryngology, University Medicine Greifswald, 17475 Greifswald, Germany; benjamin.fenske@med.uni-greifswald.de (B.F.); thomas.bremert@med.uni-greifswald.de (T.B.); christian.scharf@med.uni-greifswald.de (C.S.); chia-jung.busch@med.uni-greifswald.de (C.-J.B.); markus.blaurock@med.uni-greifswald.de (M.B.); 2 Institute of Bioinformatics, University Medicine Greifswald, 17475 Greifswald, Germany; marcus.vollmer@med.uni-greifswald.de

**Keywords:** tumor, sleep-related breathing disorders, head and neck cancer, obstructive sleep apnea

## Abstract

*Background and Objectives*: Obstructive sleep apnea (OSA) is common not only in the general population but even more so in patients with tumors of the head and neck region. Untreated, it leads to reduced quality of life, increased daytime sleepiness, and other comorbidities. The aim of this study was to determine the difference in the occurrence of OSA in the patient population with head and neck tumors compared with the general population as represented by the Trend cohort of the Study of Health in Pomerania (SHIP), and to assess the influence of tumor treatment. *Materials and Methods*: Between July 2018 and December 2021, preoperative polysomnography was conducted in 47 patients with histologically confirmed squamous cell carcinoma in the oropharynx, hypopharynx, or larynx. A follow-up polysomnography was performed in 23 patients 2–11 months after completing treatment. The collected data were correlated with tumor treatment and tumor size. *Results:* Of the included patients, 43 were male and 4 were female. Age ranged from 54 to 90 years. The pretherapeutic measurement found no significant difference in the prevalence of a pathologically elevated apnea–hypopnea index (AHI) in our patients compared with the SHIP Trend cohort. In the follow-up measurement after completion of treartment, a significant deterioration in AHI was observed. Initially, 70% of patients had an AHI > 5; after therapy, this increased to 87% (*p* = 0.008). The effect was particularly pronounced in the group of patients with advanced tumor stages who had received primary chemoradiation. *Conclusions*: OSA is a relevant condition in patients with head and neck cancer. Tumor treatment can lead to an increased occurrence of sleep-related breathing disorders, especially in patients with advanced tumor stages undergoing primary chemoradiation. Additional studies are necessary to better understand the exact mechanism involved.

## 1. Introduction

Sleep-related breathing disorders are a common condition in the general population, with obstructive sleep apnea (OSA) being particularly prevalent [1]. They are caused by a collapse of the upper airway during sleep, resulting in partial or complete airway obstruction with apnea and hypopnea. Symptoms of OSA include rhonchopathy, daytime sleepiness leading to involuntary sleeping, reduced cognitive performance, and reduced quality of life. Additionally, individuals with OSA may experience insomnia, nocturnal palpitations, nocturia, cephalgia, and depressive disorders [2]. The diagnosis and subsequent treatment of OSA are important due to the long-term consequences and complications associated with this condition, such as depression, metabolic syndrome, atherosclerosis, hypertension, stroke, and arrhythmia. Two large population-based cross-sectional studies demonstrated an odds ratio of 1.42–1.72 for the presence of arterial hypertension in subjects with an AHI > 15 compared to those without OSA [3,4]. Other epidemiological studies have shown an increased risk of cardiovascular diseases. In the Sleep Heart Health Study, there was an odds ratio of 1.58 for stroke in subjects with an AHI > 11 compared with those with an AHI ≤ 1.3, corresponding to those without OSA [5]. The increased mortality from cardiac events in patients with untreated severe OSA compared with treated patients is most likely to be attributable to more frequently occurring cardiovascular changes [6]. Early diagnosis and treatment of OSA can reduce the risk of cardiac disease and associated symptoms [7]. Therefore, OSA is a condition associated with significant health and socioeconomic consequences that should be identified and treated early.

In the population-based Study of Health in Pomerania (SHIP–Trend), the population of northeast Germany was examined to determine health-related risk factors and clinical diseases and how these change over the long term. Among other tests, polysomnography was conducted on 1264 participants. It was found that 46% of the participants had an AHI (apnea–hypopnea index) greater than 5, with 59% of men and 33% of women being affected. The AHI was highest in the group of men over the age of 60 [8]. Other studies have also found a positive correlation between age and gender. It was found that OSA was particularly common in the population of older men. Additional risk factors for the occurrence of OSA include arterial hypertension, diabetes mellitus, and obesity [9,10,11].

Head and neck tumors and their treatment are thought to be an independent risk factor for the occurrence of sleep-related breathing disorders. Several studies have been conducted based on the assumption of increased breathing disorders in this patient population. In the literature, post-therapeutic prevalence of OSA has been reported to range between 25% and 88% [12,13]. The aim of this study was to determine the prevalence of OSA in head and neck tumor patients before and after treatment, adjusted by tumor site, size, and treatment modality. The SHIP–Trend study, based on the same study site and population as our cancer center, allowed for a uniquely suited reference population to compare with our cohort.

## 2. Materials and Methods

### 2.1. Participants and Recruitment

The study was reviewed and approved by the Ethics Committee of the Greifswald University Hospital under internal registration number BB193/17 on 29 December 2017. Informed consent was obtained from all patients prior to participation. Oncological treatment was not influenced by the study, but patients received appointments for continuous positive airway pressure treatment (CPAP) evaluation if they had clinically relevant OSA at the second visit. The scope of the study was determined prior to initiation based on an estimated prevalence of OSA (obstructive sleep apnea) in the head and neck tumor patient group at 15–20% and in the general population at 7%. Following the ‘test of equal given proportions’, a sample size of 40 patients was calculated to achieve a significance level of *p* < 0.05. In total, 47 patients with histologically confirmed head and neck squamous cell carcinoma (HNSCC) of the oropharynx, hypopharynx, and larynx were recruited between July 2018 and December 2021 in the Department of Otorhinolaryngology, Greifswald University Hospital, Germany. Considering that treatment should result in a significant alteration of the upper airways, only laryngeal carcinomas with a T stage ≥ 2 were included. For oropharyngeal and hypopharyngeal tumors, a T1 stage was sufficient for participation in the study. Patients with previous head and neck tumors were excluded. Secondary exclusion criteria included tracheostomy, laryngectomy, tumor recurrence, residual disease, second primary carcinoma, and palliative treatment approach. A follow-up examination was conducted 3–8 months after the completion of tumor therapy. During the second measurement, polygraphy was performed with the parameters mentioned above, and BMI and thyroid function were determined once again.

Patient data collected included age, gender, BMI, nicotine consumption, and thyroid dysfunction. Daytime sleepiness was assessed using the Epworth sleepiness scale (ESS).

### 2.2. Scoring of Respiration

While polysomnography would have been potentially beneficial, it was not practical in our setting and would have put significantly more burden on the patients, prolonging their inpatient stay. As part of the staging examinations at the time of diagnosis, patients underwent overnight polygraphy using a MiniScreen Plus 4-channel device from Löwenstein Medical GmbH & Co. KG. (Bad Ems, Germany). The polygraphy recorded body position, thoracic and abdominal respiratory movements via chest and abdominal belts with integrated pressure pads, oxygen saturation (SpO2), and heart rate, using a pulse oximeter. Additionally, airflow was measured using a nasal cannula with a length of 20 cm (REF: 200-0312/01 Löwenstein) for pressure measurement. 

The analysis of the polygraphy data was performed using the MiniScreen Viewer (Version 5.17bR5, (Bad Ems, Germany) following criteria established by the American Academy of Sleep Medicine (AASM) in 2012. Apnea events were defined as lasting at least 10 s and showing a reduction in airflow of more than 90%. Hypopnea events were defined as respiratory disturbances lasting at least 10 s, with a reduction in airflow between 30% and 90%, and a decrease in oxygen saturation of at least 3% compared with the preceding arterial oxygen saturation.

Regarding the polysomnography performed in the SHIP–Trend study, the methodology is published in Fietze, I., et al. [8]. 

### 2.3. Statistical Analyses

Statistical analysis was carried out using IBM SPSS 27 Statistics. A *p*-value less than 0.05 was considered statistically significant. Due to the small size of the patient population in the dependent samples, the Wilcoxon signed-rank test was used, and for independent samples, the Mann–Whitney U test was employed for statistical assessment. The relationship between the POTT study and the SHIP–Trend study was assessed using the Chi-squared test. Potential treatment effects on BMI were evaluated with a paired *t*-test.

## 3. Results

### 3.1. Pre-Therapeutic Prevalence of Sleep-Related Breathing Disorders (OSA) in the Head and Neck Tumor Group Compared with the General Population

A total of 47 patients were included in the study, based on the criteria outlined above. Among them, 43 were male, and 4 were female. The age ranged from 54 to 90 years, with an average age of 63.4 and a standard deviation of 6.6. Out of the 47 patients, 34 were over 60 years old, with 33 being male and 1 female. There were only 13 patients under 60 years old, 10 males and 3 females.

Tumor staging was classified according to the eighth edition of the TNM classification. There were 3 T1 tumors (6%), 12 T2 tumors (25.5%), 8 T3 tumors (17%), and 24 T4 tumors (47%) diagnosed. The specific tumor locations are listed in Table 1.

Out of the 47 patients, 33 (70%) had an AHI greater than or equal to 5. Among the 33 patients, 20 (60%) had an AHI between 5 and <15, 9 (27%) had an AHI between 15 and <30, and 4 (12%) had an AHI ≥ 30. The average AHI among these patients was 13.3, with a standard deviation of 14.5.

In the age group under 60 years, 8 (13%) of 13 patients had an AHI ≥ 5. Among these 8 patients, 5 (63%) had an AHI between 5 and <15, 2 (25%) had an AHI between 15 and <30, and 1 (13%) had an AHI ≥ 30. The average AHI in this group was 13.22, with a standard deviation of 14.6.

In the age group over 60 years, 25 (73%) of 34 patients had an AHI ≥ 5. Among these 25 patients, 15 (60%) had an AHI between 5 and <15, 7 (28%) had an AHI between 15 and <30, and 3 (12%) had an AHI ≥ 30. The average AHI in this group was 13.6, with a standard deviation of 14.6. There was no significant difference in AHI between the two age groups (Mann–Whitney U Test *p* = 0.62).

**Figure 1:**
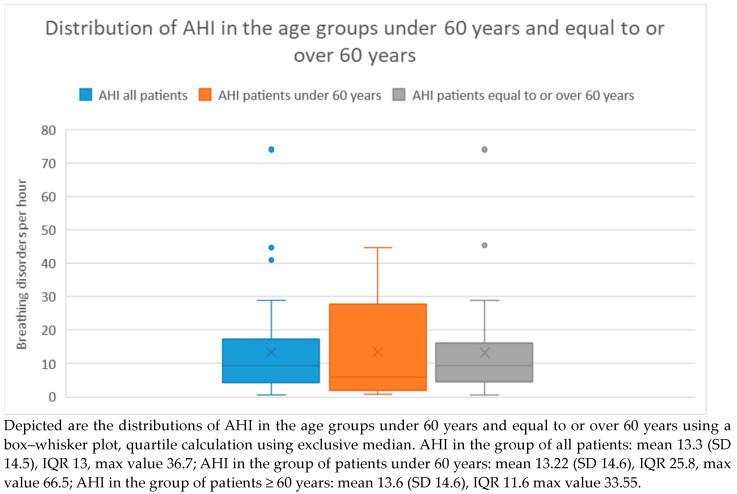
Age-group-related distribution of AHI.

The distribution of AHI severity categories is presented in Table 2 and Figure 1 illustrates the distribution of AHIs in the different groups using a box plot.

The analysis of the ESS questionnaire yielded an average score of 4.7, with a standard deviation of 3.5.

The SHIP–Trend Study was chosen as a reference population to our study cohort. It is based in the same city and examines the same population of West Pommerania that our cancer center treats. The SHIP–Trend study was conducted between 2008 and 2012; 1208 probands were examined and evaluated using polysomnography according to the AASM criteria from 2007. Table 2 presents the probands from the SHIP–Trend study and the patients from the POTT study based on the severity of AHI. In the Chi-squared test, no significant difference was found between head and neck tumor patients over 60 years old and the general population (*p* = 0.58). Similarly, in patients under 60 years old, no significant difference was found compared with the general population (*p* = 0.12).

### 3.2. Change in AHI (Apnea–Hypopnea Index) in the Head and Neck Tumor Group over the Course of Treatment

The second study visit following cancer treatment was performed in 23 out of the 47 patients initially recruited. The follow-up occurred between the 2nd and 11th month following the completion of tumor treatment, with an average of 5 months (standard deviation 2.3). Among the 23 patients, 20 were male, and 3 were female. In total, 13 patients were over 60 years old, while 10 were under 60 years old.

Among these 23 patients, 12 received primary chemoradiation and the remaining 11 underwent surgery followed by adjuvant radiotherapy or chemoradiation.

Tumor staging revealed three T1 tumors (13%), seven T2 tumors (30%), four T3 tumors (17%), and nine T4 tumors (39%). The specific tumor locations are listed in Table 3.

When considering all patients, the pre-therapeutic AHI ranged from 0.6 to 41 with a mean of 11.6 and a standard deviation of 10.3. Post-therapeutically, the AHI ranged from 0.8 to 63.1 with a mean of 19.1 and a standard deviation of 15.8. Regarding the difference in AHI between pre- and post-therapeutic measurements, the minimum was −40.2 and the maximum was 45.9, with a mean of 7.5 and a standard deviation of 16.7.

**Figure 2:**
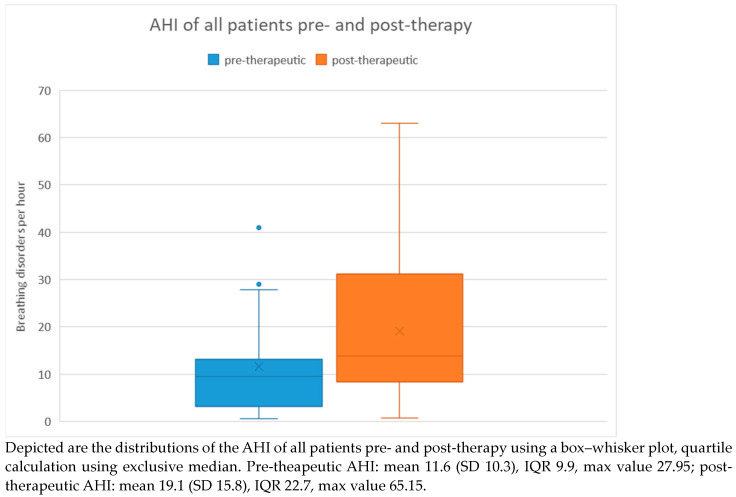
AHI of all patients prior to and after tumor therapy.

Statistical analysis using the Wilcoxon signed-rank test revealed a significant difference (*p* = 0.008) between pre- and post-therapeutic measurements. This is further illustrated in Figure 2 using a box plot.

**Figure 3:**
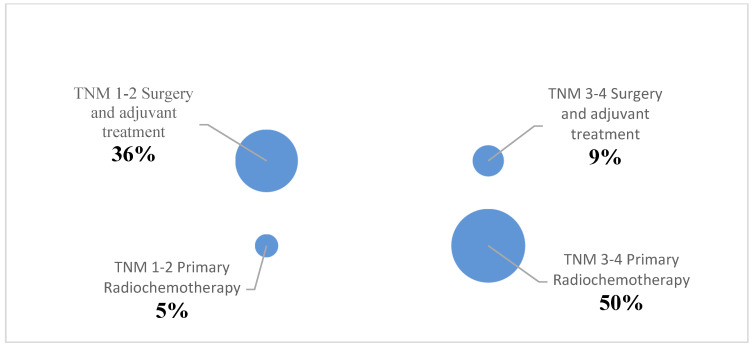
Distribution of patients based on type of treatment and tumor size.

In terms of the severity of AHI, among the 23 patients, 16 (70%) had an AHI ≥ 5 pre-therapeutically. Among these 16 patients, 11 (69%) had an AHI between 5 and <15, 4 (25%) between 15 and <30, and 1 (6%) had an AHI ≥ 30. After treatment, 90% of the same 23 patients had an AHI ≥5. Among the 20 affected individuals, 11 (55%) had an AHI between 5 and <15, 3 (15%) between 15 and <30, and 6 (30%) had an AHI ≥ 30. Table 4 and Figure 3 provide a breakdown based on the severity of AHI.

Due to the number of patients in the second part of this study, the statistical analysis was divided into two groups. Initially, patients were divided into the groups T1/T2 tumors and T3/T4 tumors, based on the primary tumor size. Ten patients were assigned to the T1/T2 tumor group, and 13 patients were assigned to the T3/T4 tumor group. Also, patients were divided based on their treatment. For this purpose, patients were categorized into groups receiving primary chemoradiation (12 patients) or surgery with adjuvant treatment (11 patients).

#### 3.2.1. BMI

The patients’ BMI values were determined before and after treatment. The mean pretherapeutic BMI was 26.2 with a standard deviation of 4.8. Post-therapeutically, the mean BMI was 23.6 with a standard deviation of 4.3. This change was highly significant in the *t*-test (*p* < 0.001).

#### 3.2.2. Comparison of AHI within the Groups with T1/T2 Tumors and T3/T4 Tumors

**Figure 4:**
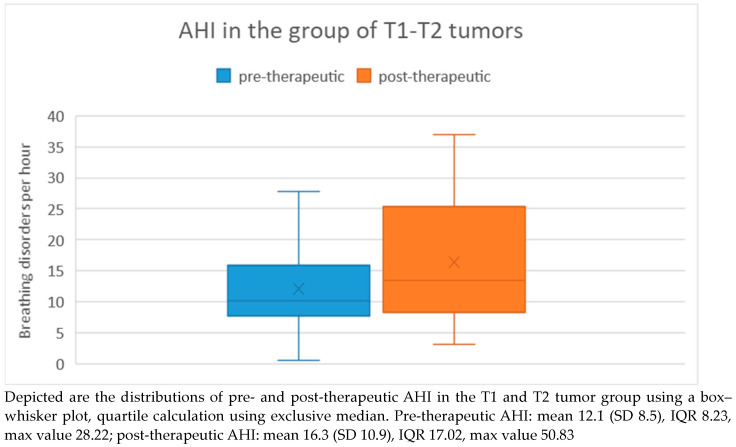
AHI in the T1/T2 tumor group.

When comparing the pre-therapeutic AHI measurements between the T1/T2 tumor group and the T3/T4 tumor group, the Mann–Whitney U test did not reveal a significant difference (*p* = 0.47). In the T1/T2 tumor group, the pre-therapeutic AHI ranged from 0.6 to 27.8 with a mean of 12.1 and a standard deviation of 8.5. Post-therapeutically, the AHI ranged from 3.1 to 37 with a mean of 16.3 and a standard deviation of 10.9. Regarding the difference in AHI between pre- and post-therapeutic measurements, the minimum was −9.3 and the maximum was 22.4, with a mean of 4.3 and a standard deviation of 9.2 (Figure 4). Statistical analysis using the Wilcoxon signed-rank test showed no significant difference (*p* = 0.20) between pre- and post-therapeutic measurements in this group.

**Figure 5:**
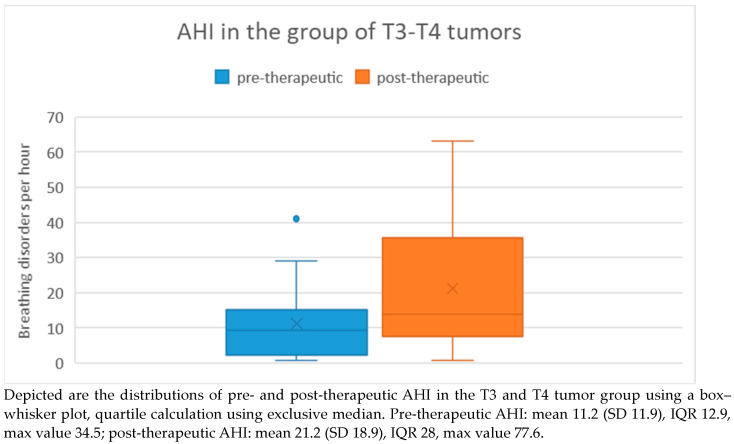
AHI in the T3/T4 tumor group.

In the T3/T4 tumor group, the pre-therapeutic AHI ranged from 0.8 to 41 with a mean of 11.2 and a standard deviation of 11.9. Post-therapeutically, the AHI ranged from 0.8 to 63.1 with a mean of 21.2 and a standard deviation of 18.9. Regarding the difference in AHI between pre- and post-therapeutic measurements, the minimum was −40.2 and the maximum was 45.9, with a mean of 10 and a standard deviation of 20.9 (Figure 5). Statistical analysis using the Wilcoxon signed-rank test revealed a significant difference (*p* = 0.019) between pre- and post-therapeutic measurements in this group.

#### 3.2.3. Comparison of AHI within the Groups Receiving Primary Chemoradiation and Surgery with Adjuvant Treatment

**Figure 6:**
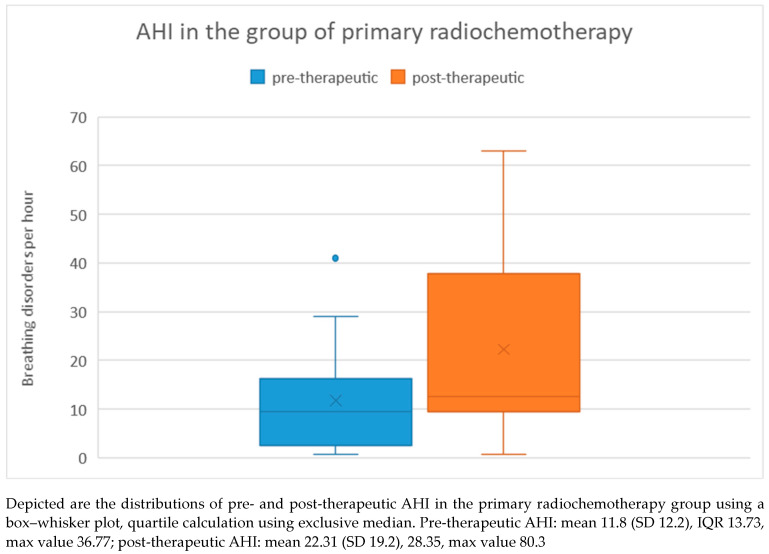
AHI in the primary chemoradiation group.

There was no significant difference in pre-therapeutic AHI between the two treatment groups, as determined by the Mann–Whitney U test (*p* = 0.83). Among patients who received primary chemoradiation, the pre-therapeutic AHI ranged from 1 to 41 with a mean of 11.8 and a standard deviation of 12.2. Post-therapeutically, the AHI ranged from 1 to 63 with a mean of 22.31 and a standard deviation of 19.2. Regarding the difference in AHI between pre- and post-therapeutic measurements, the minimum was −40.2 and the maximum was 45.9, with a mean of 10.5 and a standard deviation of 21.7 (Figure 6). Statistical analysis using the Wilcoxon signed-rank test showed a significant difference (*p* = 0.028) between pre- and post-therapeutic measurements in this group.

**Figure 7:**
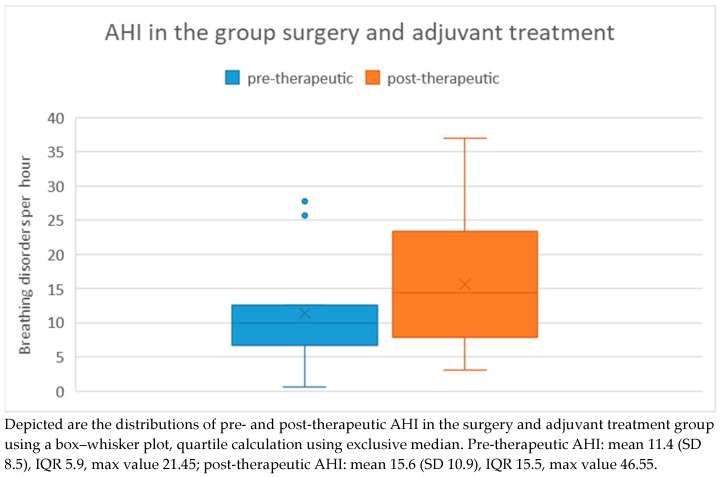
AHI in the group with surgery and adjuvant treatment.

Among patients who underwent surgical treatment with subsequent adjuvant therapy, the pre-therapeutic AHI ranged from 1 to 28 with a mean of 11.4 and a standard deviation of 8.5. Post-therapeutically, the AHI ranged from 3 to 37 with a mean of 15.6 and a standard deviation of 10.9. Regarding the difference in AHI between pre- and post-therapeutic measurements, the minimum was −9.3 and the maximum was 22.4, with a mean of 4.2 and a standard deviation of 8.8 (Figure 7). Statistical analysis using the Wilcoxon signed-rank test did not reveal a significant difference (*p* = 0.182) between pre- and post-therapeutic measurements in this group.

## 4. Discussion

Sleep-related breathing disorders are not only common in the general population but are also frequently found in head and neck cancer patients [1,12,13,14].The aim of this study was to determine whether there is a difference in sleep-related breathing disorders between patients with head and neck tumors and an age-adjusted cohort of the general population. We also wanted to assess the impact of treatment on these sleep-related breathing disorders. Untreated obstructive sleep apnea not only has negative health consequences but also leads to reduced quality of life and worse outcomes following treatment for head and neck cancer [2,5,15]. Previous studies have shown that there may be an increased prevalence of such breathing disorders, especially in the head and neck cancer patient group [12,13]. However, it has not been definitively shown whether patients already have a pathologically elevated AHI before undergoing treatment or whether it worsens during the course of therapy.

Comparison of the currently available studies is challenging for several reasons, including changes in recent years in the criteria used to evaluate sleep-related breathing disorders via polysomnography or polygraphy. The criteria set by the American Academy of Sleep Medicine (AASM) in 2007 were stricter than the current guidelines, which could lead to an increased prevalence in today’s studies. For example, the HypnoLaus study was evaluated using both the 2012 and 2007 criteria, resulting in a decrease in the median AHI from 9.9 to 4.3 [16].

Furthermore, some studies differentiate between obstructive apnea (OSA) and obstructive sleep apnea–hypopnea syndrome (OSAHS). The definitions of these terms are not always consistent and often overlap. Some authors use the term OSA when there are breathing disturbances and reserve the term OSAHS for cases where daytime sleepiness is also present in addition to OSA. This approach partially contradicts the internationally accepted definition in the International Classification of Sleep Disorders (ICSD-3). Due to the issues related to inconsistent definitions and usage of the terms OSA and OSAHS, they may not be suitable as a comparative benchmark between studies [8,16,17].

Additionally, the age and gender distribution of the participants included in the studies often vary. For example, the Wisconsin Sleep Cohort Study examined only adults aged 30 to 60 years, finding an AHI > 5 in 24% of men and 9% of women [18]. In contrast, the SHIP–Trend study reported an AHI of 5 or higher in 67% of participants over the age of 60 [8].

The prevalence of OSA in head and neck cancer patients varies in the current literature, and this variability can be attributed to differences in study designs, criteria for evaluation, technical methods for assessing sleep-related breathing disorders, and the heterogeneity of tumor diseases and their treatments. Studies that examined head and neck cancer patients before treatment reported a range of patients with an AHI ≥ 5, between 50–90% [14,15]. With a prevalence of 70% (33 out of 47 patients) having an AHI ≥ 5, our study falls within the middle of this range. In a direct comparison with the age-related general population from the SHIP–Trend study, there was no significant difference in either the under-60 or over-60 age groups in the POTT study. However, it is important to note that the SHIP–Trend study applied the AASM guidelines from 2007, while the current study used the 2012 guidelines. In the AASM criteria from 2007, hypopnea is defined as a reduction in airflow of 30% or more for at least 10 s along with a minimum 4% drop in oxygen saturation. In the AASM criteria from 2012, hypopnea is defined with a 3% drop in oxygen saturation. The criteria for scoring apnea remain the same. This difference in the required drop in oxygen saturation leads to a significant change in the AHI when evaluating sleep-related breathing disorders. This difference in guidelines could potentially lead to a slightly higher prevalence in our patient cohort.

Another important consideration is that sleep-related breathing disorders were assessed using polygraphy in our study and polysomnography in the SHIP–Trend study. Since polygraphy does not detect waking episodes during recording, it may lead to a reduction in the actual AHI compared with polysomnography. Nevertheless, despite these differences, the SHIP–Trend study appears to be the most suitable study for comparison with our patient cohort due to its scope, study design, and data presentation.

In the majority of studies, patients who had already received tumor treatment were examined. In a study conducted by Huppertz et al. in 2020, 94% of the 17 patients examined had an AHI ≥ 5 [15]. A French study from 2017 found that 25% of their 51 included patients had an AHI ≥ 10 [13]. Another study from 2009 with 31 patients found that 19% of them had an AHI ≥ 20 [19]. In our study, the AHI was above 5 in 87% of the 23 post-treatment patients and above 15 in 39% of the patients. Due to differences in study designs, the heterogeneity of tumor diseases and their treatments, and different definitions of OSA, a direct comparison between studies is challenging. Overall, our study seems to fall within the higher range of estimates for OSA prevalence.

Different treatment strategies have varying effects on nocturnal breathing disorders. Colleagues from Taiwan conducted a study in 2021 to examine how head and neck cancer patients’ AHI changes immediately after surgery. In that study, 15 patients underwent pre- and post-operative polygraphy. The post-operative measurement was taken on average 154 days after the operation and before any adjuvant treatment. No significant difference was found between the pre- and post-operative AHI [20]. In a 2009 study conducted in Germany, 31 patients underwent polygraphy after tumor resection. There was no significant influence in the presence of OSA following radiation therapy. However, the study did not use AHI as the comparison metric but defined OSA as having an AHI ≥ 20. Additionally, only patients with smaller tumors (T1–T3) were included [19]. In the French study conducted by A. Loth, where 51 patients were examined for the presence of OSA after tumor treatment, no significant difference was found between individuals who received primary radiochemotherapy and those who underwent surgery with adjuvant treatment [13]. However, the study by W. Qian yielded different results: of the 24 included patients, only 33% of those who received primary radiochemotherapy had an AHI ≥ 15, whereas 75% of those who underwent surgery with adjuvant therapy did. It should be noted, however, that the group receiving primary radiochemotherapy predominantly consisted of patients with oropharyngeal carcinoma (5/9), while the group undergoing surgery primarily comprised patients with oral cavity carcinoma (14/15). In patients who underwent surgery, the defect was reconstructed using a microvascular radial flap. The resulting volume increase and altered anatomy in the oral cavity region may have complicated a direct comparison between the groups [21]. In summary, the currently available studies provide a heterogeneous picture on this issue, and the question cannot be conclusively answered.

Regarding the influence of tumor size on AHI, there are also conflicting reports in the literature. Huppertz et al. reported that a post-treatment increase in AHI was mainly observed with smaller tumors [15]. However, Huyett et al. found no significant influence of tumor size and treatment on AHI [14]. Similarly, studies by Loth et al. [13] and Qian et al. [21] did not find evidence of a correlation between tumor size and OSA.

In our study, when considering the influence of treatment type and tumor size, patients were divided into two groups: (i) patients with smaller tumors who underwent surgery with subsequent adjuvant treatment, and (ii) patients with larger tumors who received primary chemoradiation. Among patients with larger tumors and primary chemoradiation, a significant change in AHI was observed. However, in the other group there was no significant change. Unfortunately, due to the distribution of our patients into these two groups and the size of the study, it is not possible to conclusively answer whether tumor size or treatment was the deciding factor.

The work by Huppertz et al. [15] is probably the most relevant comparative study regarding the question of the significance of tumor size and treatment. The study design was very similar to that of the POTT study. A total of 33 patients were included, of whom 17 underwent post-treatment follow-up. Patients were classified according to UICC stages. The colleagues concluded that there was no deterioration in AHI between pre- and post-treatment measurements across the entire patient cohort. However, there was a slight improvement in AHI in UICC stages III and IV, while a deterioration occurred in UICC stages I and II. Nevertheless, there are some differences between the studies that make a direct comparison challenging. One limitation of the UICC stage classification is that smaller tumors can be classified into higher stages due to positive lymph node findings or distant metastases. Furthermore, the study predominantly included patients with smaller tumors (59% UICC stages I and II). In the POTT study, on the other hand, 57% of the tumors were T3 or T4 stage. Patients with UICC stage IV in Huppertz et al.’s study were all treated surgically, while a majority of T3 and T4 tumors in our study received primary chemoradiation. Although the two studies come to different conclusions, this does not necessarily mean they are in direct conflict. Assuming that resection of larger tumors may lead to a greater effect on upper airway expansion, a corresponding deterioration in AHI due to primary chemoradiation seems plausible. Given that the majority of patients in our cohort, especially those with T3 and T4 stage tumors, received primary chemoradiation, the deterioration in AHI in this group is understandable and aligns with the assumption mentioned above. The high proportion of T3 and T4 tumors in the POTT study could also explain why there is a significant difference between pre- and post-treatment measurements in that study, whereas in Huppertz et al.’s study, there was no significant difference.

## 5. Conclusions and Outlook

Sleep-related breathing disorders are a relevant medical condition for head and neck tumor patients. With regard to our patient cohort, it can be stated that there was a deterioration over the course of tumor treatment. This deterioration was particularly pronounced in the group with a higher tumor stage and primary chemoradiation. Therefore, in daily clinical practice, risk factors should be assessed in these patients, and indication for further diagnostics and treatment should be considered if necessary. 

Our study and the studies before it suffer from similar limitations concerning sample size and heterogeneity. The high dropout rate is very typical and seen across studies. A multicenter study with clearly defined subgroups should be attempted. If possible, this should include polysomnography and sleep endoscopy, and possibly volumetric analysis of the airways during the course of treatment. The funding of a large multicenter study is a significant hurdle and we hope that our work along with the studies cited can lay the foundation for this process.

## Figures and Tables

**Table 1:** Subdivision of all pre-therapeutic patients based on tumor size and location.

	T1	T2	T3	T4	Total
Hypopharyngeal carcinoma	1 (2%)	3 (6%)	2 (4%)	6 (12%)	12 (25%)
Oro-hypopharyngeal carcinoma	0	0	0	4 (9%)	4 (9%)
Oropharyngeal carcinoma	2 (4%)	7 (15%)	3 (6%)	8 (17%)	20 (42%)
Laryngeal carcinoma	0	2 (4%)	1 (2%)	3 (6%)	6 (12%)
Hypopharynx–larynx carcinoma	0	0	2 (4%)	3 (6%)	5 (10%)
Total	3 (6%)	12 (26%)	8 (17%)	24 (47%)	47

**Table 2:** The division of participants in the POTT study and the SHIP–Trend study based on AHI severity levels.

	SHIP–Trend in Total (n = 1208)	SHIP–Trend under 60 Years (n = 796)	SHIP–Trend over 60 Years (n = 412)	POTT Study in Total (n = 47)	POTT Study under 60 Years (n = 13)	POTT Study over 60 Years (n = 34)
AHI 0–<5	604 (50%)	476 (60%)	128 (31%)	14 (30%)	5 (38%)	9 (26%)
AHI 5–<15	317 (26%)	188 (24%)	129 (31%)	20 (42%)	5 (38%)	15 (44%)
AHI 15–<30	182 (15%)	88 (11%)	94 (23%)	9 (19%)	2 (15%)	7 (20%)
AHI ≥ 30	105 (9%)	44 (5%)	61 (14%)	4 (9%)	1 (8%)	3 (9%)
mean ± SD	10.7 ± 14.3	8.1 ± 12.3	15.7 ± 16.5	13.3 ± 14.5	13.6 ± 14.6	13.2 ± 14.6

**Table 3:** Subdivision of all post-therapeutic patients based on tumor size and location.

	T1	T2	T3	T4	Total
Hypopharyngeal carcinoma	1 (4%)	1 (4%)	0	1 (4%)	3 (13%)
Oro-hypopharyngeal carcinoma	0	0	0	3 (13%)	3 (13%)
Oropharyngeal carcinoma	2 (9%)	3 (13%)	2 (9%)	3 (13%)	10 (43%)
Laryngeal carcinoma	0	3 (13%)	1 (4%)	2 (9%)	6 (26%)
Hypopharynx–larynx carcinoma	0	0	1 (4%)	0	1 (4%)
Total	3 (13%)	7 (30%)	4 (17%)	9 (39%)	23

**Table 4:** Presentation of AHI severity levels in patients who participated in both visits.

	Pre-Therapeutic	Post-Therapeutic
AHI 0–<5	7 (30%)	3 (13%)
AHI 5–<15	11 (47%)	11 (47%)
AHI 15–<30	4 (17%)	3 (13%)
AHI ≥ 30	1 (4%)	6 (26%)
mean ± SD	11.6 ± 10.3	19.1 ± 15.8

## Data Availability

Due to local data protection guidelines regarding data from clinical trials, the raw data can only be made available after reasonable request to the corresponding author. This request will be evaluated by the local IRB.
